# The Hospital Association Conference

**Published:** 1923-05

**Authors:** 


### BRITISH HOSPITALS' ASSOCIATION.

### THE SHEFFIELD CONFERENCE.

The annual conference of the British Hospitals Association is to be held at Sheffield on Thursday and Friday, May 31 and June 1. The meeting will take place at the Victoria Station Hotel and the President will be the Hon. Sir Arthur Stanley, G.B.E. The first business of the Conference will be to consider the reference from the last annual meeting concerning the establishment of a central fund for the provincial hospitals. An address will be given by Mr. H. J. Waring, F.R.C.S., Vice-Chancellor of London University (Hon. Surgeon, St. Bartholomew's Hospital), on "The Future of Voluntary Hospitals from a Medical View-point." The address will be followed by discussion. At 2.15 p.m. an address, on "Modern Hospital Administration," will be followed by a discussion, to be opened by Dr. S. S. Goldwater (Mount Sinai Hospital, New York). Later in the afternoon there will be a reception at the Town Hall by the Lord Mayor of Sheffield and Sir Henry Hadow, Vice-Chancellor of Sheffield University and Chairman of the Sheffield Joint Hospital Council, and in the evening an official dinner to the delegates at the Royal Victoria Hotel, when the Chairman will be Sir Henry Hadow.

#### Visits to Hospitals.

The following day at 10 a.m. a business meeting to elect honorary officers and Council and to fix time and place of the fourteenth annual conference will be held, followed by a statement by Sir Napier Burnett on "Hospital Finance." Lord Hambleden will then speak on "Hospital Finance in its Relation to Approved Societies," and his address will be followed by a discussion. A luncheon at 1 p.m. at the Cutlers' Hall will be given by the Master Cutler (Mr. R. W. Matthews). The following hospitals and convalescent homes will be open to delegates from 2.30 p.m. to 4.30 p.m.: Sheffield Royal Infirmary; Sheffield Royal Hospital; Jessop Hospital for Women; Children's Hospital; Edgar Allen Institute; King Edward VII. Hospital (Municipal), Children's Open Air Hospital; Fir Vale Hospital (Municipal); Royal Hospital Convalescent Annexe; George Woofinden Convalescent Home; and the Sheffield Works Convalescent Home.

#### Hospitality Arrangements.

The Conference Committee will be pleased to provide private hospitality for those delegates who do not prefer to secure their own hotel accommodation. Delegates will be met and taken to their respective hosts if they will notify the Regional Secretary beforehand in good time as to the day and hour of their arrival and the route by which they are travelling. The Guest House at Froggatt, near Grindleford, amidst typical Derbyshire scenery, has been placed at the disposal of the delegates for the week-end following the Conference, at a nominal cost of 10s. 6d. per day, inclusive: dinner, bed, breakfast, luncheon, and tea. Official programmes for the conference will shortly be available and can be obtained from the Regional Secretary, Mr. S. R. Lamb, St. Peter's Close, Hartstead, Sheffield.

